# Hospital mortality in acute coronary syndrome: adjustment of GRACE score by D-dimer enables a more accurate prediction in a prospective cohort study

**DOI:** 10.1186/s12872-019-1239-4

**Published:** 2019-11-10

**Authors:** Tongtong Yu, Yundi Jiao, Jia Song, Dongxu He, Jiake Wu, Zhijun Sun, Zhaoqing Sun

**Affiliations:** Department of Cardiology, Shengjing Hospital of China Medical University, Shenyang, Liaoning People’s Republic of China

**Keywords:** D-dimer, In-hospital mortality, Acute coronary syndrome, GRACE score, Percutaneous coronary intervention

## Abstract

**Backgroud:**

To assess the value of D-dimer and its combination with The Global Registry of Acute Coronary Events (GRACE) score in predicting in-hospital mortality in patients with acute coronary syndrome (ACS) undergoing percutaneous coronary intervention (PCI).

**Methods:**

In 5923 ACS patients undergoing PCI, the role of D-dimer and the added value of D-dimer to GRACE score for predicting in-hospital mortality were tested.

**Results:**

After multivariable adjustment, D-dimer could significantly predict in-hospital mortality. Also, it could significantly improve the prognostic performance of GRACE score (C-statistic: *z* = 2.269, *p* = 0.023; IDI: 0.016, *p* = 0.032; NRI: 0.291, *p* = 0.035).

**Conclusion:**

In patients with ACS undergoing PCI, D-dimer was an independent predictor of in-hospital death. It could also improve the prognostic performance of GRACE score.

## Background

Acute coronary syndrome (ACS) patients still have a poor prognosis, even receiving timely percutaneous coronary intervention (PCI) and/or adequate antiplatelet drugs [1–4]. Early risk stratification is essential for making clinical decision and evaluating prognosis. D-dimer is a kind of degradation product of fibrin [5]. It is also a biomarker of coagulation state and the form of thrombosis [5]. Elevated D-dimer was associated with vulnerable plaque [6], no-reflow after PCI [7, 8] and a larger myocardial injury assessed by cardiac magnetic resonance (CMR) [9]. Many studies also confirmed the association between higher D-dimer and the increased long-term mortality in patients with stable coronary artery disease [10, 11], ACS [12] and ST-segment elevation myocardial infarction (STEMI) [13, 14]. However, it isn’t well known about the role of D-dimer in predicting in-hospital mortality in ACS patients.

The Global Registry of Acute Coronary Events (GRACE) score is recommended to get in-hospital mortality in ACS patients and can help us make clinical decision and discriminate the patients on high-risk [1–4]. But, GRACE score doesn’t include new risk factors, such as D-dimer. Whether adjustment of the GRACE risk score by D-dimer will enable a more accurate prediction is not also defined.

This study tried to confirm whether D-dimer could predict in-hospital mortality, and improve the prognostic performance of the GRACE score in patients with ACS undergoing PCI.

## Methods

### Study design and setting

This study included the consecutive patients with ACS undergoing PCI at a large-scale hospital in Northeast China (Shengjing Hospital of China Medical University, Shenyang, China) from January 1, 2010 to December 31, 2017. The investigators got clinical and procedural data of all cases from electronic medical records and Picture Archiving and Communication Systems. GRACE score was gained as defined previously [1–4]. Venous blood samples were drawn from all cases on admission and measured for D-dimer using latex agglutination assays by an automatic coagulation analyzer (ACL TOP, BECKMAN COULTER, USA) in Shengjing Hospital Core Laboratory. The reference interval of D-dimer was 0–252 ng/mL. In-hospital death was defined previously [15]. Exclusion criteria included (1) use of erythropoietin, oral anticoagulants and thrombolysis (75 cases); (2) severe infections, end-stage liver or renal failure (221 cases); (3) known autoimmune diseases or steroid therapy, known malignancy, recent ischemic or hemorrhagic disease (32 cases); (4) recently undergone surgical or invasive procedures (12 cases); (5) samples collected within 5 h after use of unfractionated heparin or 12 h after use of low molecular weight heparin (LMWH) (187 cases); (6) GRACE score data missing (41 cases). At last, 5923 patients with ACS undergoing PCI were included in this study. They were then divided into three groups according to the tertile of D-dimer level (Low D-dimer group: ≤88 ng/mL [*n* = 1975]; Intermediate D-dimer group: 89–179 ng/mL) [*n* = 1974]; High D-dimer group:>179 ng/mL [*n* = 1974]). This study complies with the Declaration of Helsinki, and Shengjing Hospital of China Medical University Research Ethics Committee approved the research protocol. Written informed consent was formally obtained from all participants.

### Statistical analysis

Quantitative variables with normal distribution were represented as mean ± standard deviation (SD) and compared with variance analysis. Quantitative variables without normal distribution were represented as median [interquartile range, IQR] and compared with Kruskal-Wallis H test. Categorical variables are presented as counts and proportions (%) and compared with chi-square test. Logistic univariate regressions were performed to evaluate predictors of mortality of all variables (Additional file 1: Table S1). A multivariate logistic regression model was used to identify independent predictors of mortality. Variables with *p* < 0.1 on univariate analysis were entered a multivariate analysis (Additional file 1: Table S1). D-dimer was analyzed as a continuous variable and a categories variable, respectively. Results were reported as odds ratios (ORs) with associated 95% confidence intervals (CIs). The predictive performance of D-dimer, GRACE score and GRACE score + D-dimer was assessed by indexes of discrimination (C-statistic), calibration (the Hosmer-Lemeshow test, the Nagelkerke-R^2^) and precision (the Brier scores). The C-statistic was compared using a nonparametric test developed by DeLong et al. [16] Each model was entered into a logistic regression model to get the individual risk probability of all-cause death, respectively. The Hosmer-Lemeshow (HL) test and the Nagelkerke-R^2^ from the regression modeling was used as an indicator of goodness-of-fit of each risk model and to assess the calibration ability of them [17]. The Brier scores of D-dimer, GRACE score and GRACE score + D-dimer were also calculated [18]. Lower Brier scores indicate better calibration [18]. We also used the absolute integrated discrimination improvement (IDI) and category-free net reclassification improvement (NRI) to evaluate improvements in risk predictions quantify [19]. All tests were two-sided, and the statistical significance was defined as *p* < 0.05. All statistical analyses calculated by the Statistical Analysis System version 9.4 (SAS, SAS Institute Inc., Cary, North Carolina, USA).

## Results

### Participants and baseline characteristics

**Fig. 1:**
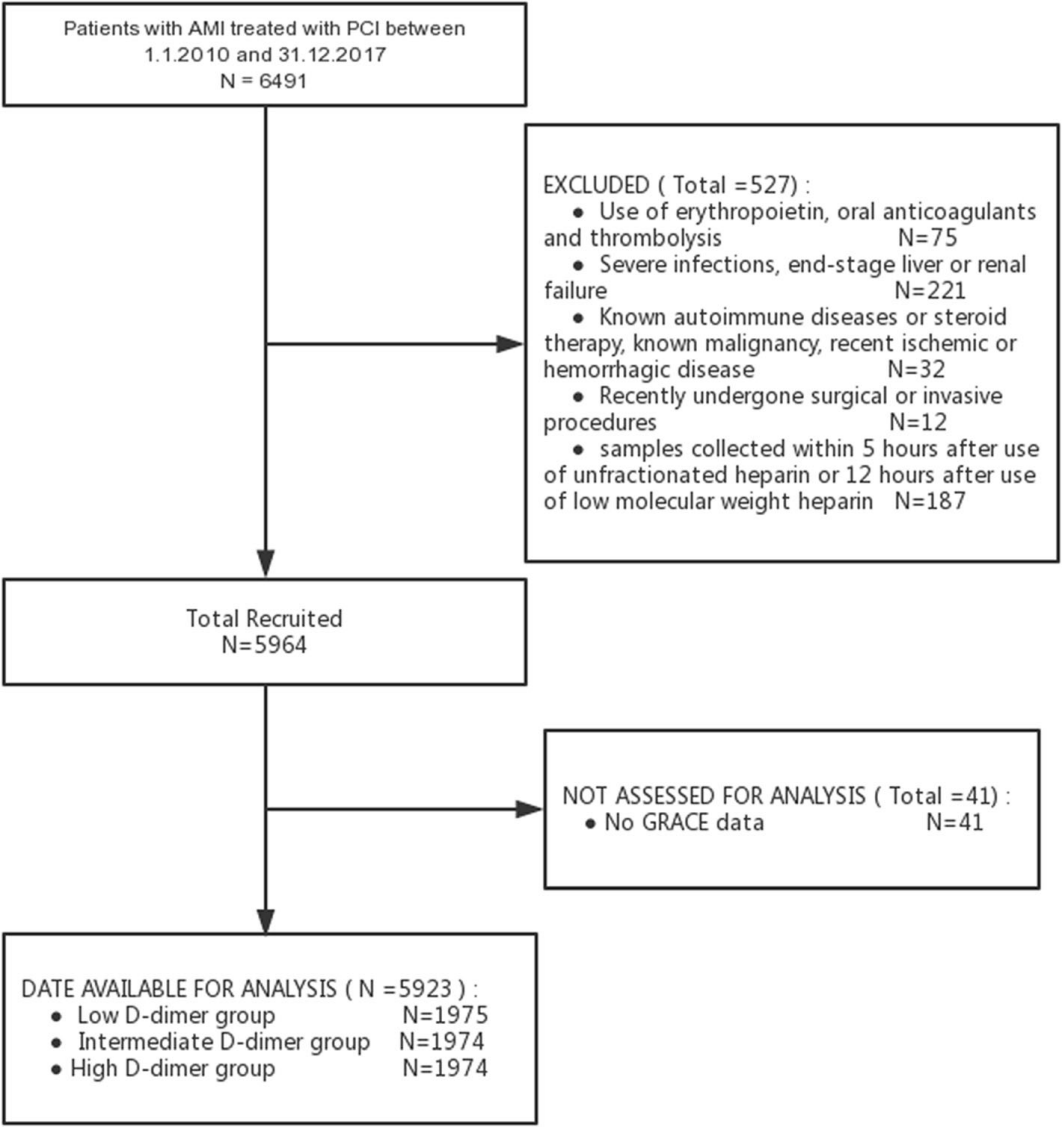
Flow diagram of participant selection

The flowchart represented the patient selection (Fig. 1). Five thousand nine hundred twenty-three patients with ACS undergoing PCI were included in the final study cohort. It was then divided into three groups: (1) Low D-dimer group [*n* = 1975]; (2) Intermediate D-dimer group [*n* = 1974]; (3) High D-dimer group [*n* = 1974]. Table 1 showed the clinical characteristics. High D-dimer group had significantly higher percentages of left main disease, females, three-vessel disease, STEMI, use of Intra-aortic Balloon Pump and TIMI grade 0/1 on arrival, compared with Low and Intermediate D-dimer group. It also had a tendency towards increasing age, heart rate, GRACE score, fibrinogen, Troponin-I, creatinine, leukocyte count and BNP on admission. The percentage of prior hypertension was significantly higher in Intermediate D-dimer group. There was a significant trend of decreasing systolic blood pressure in High D-dimer group. High D-dimer group also had a trend to have a higher in-hospital mortality (1.9% vs 0.5 and 0.3%, *p*<0.001) (Table 1).

**Table 1:** Baseline Characteristics of the study population, median (IQR), or N (%), or means±SD

Variable	Overall (*n* = 5923)	Low D-dimer group (*n* = 1975)	Intermediate D-dimer group (*n* = 1974)	High D-dimer group(*n* = 1974)	*p*-value
Demographics					
Age, yrs	62.2 ± 11.3	58.4 ± 10.2	62.9 ± 10.5	65.2 ± 12.1	<0.001
male	4117 (69.5)	1495 (75.7)	1343 (68.0)	1279 (64.8)	<0.001
Medical history					
History of Diabetes Mellitus	1875 (31.7)	595 (30.1)	630 (31.9)	650 (32.9)	0.159
History of Hypertension	3517 (59.4)	1116 (56.5)	1228 (62.2)	1173 (59.4)	0.001
History of MI	563 (9.5)	169 (8.6)	199 (10.1)	195 (9.9)	0.208
Dyslipidemia	3992 (67.4)	1369 (69.3)	1324 (67.1)	1299 (65.8)	0.058
Prior PCI	627 (10.6)	193 (9.8)	225 (11.4)	209 (10.6)	0.252
Clinical Presentation					
SBP on admission, mm Hg	135.2 ± 21.9	134.9 ± 21.1	136.4 ± 21.8	134.3 ± 22.9	0.004
Heart rate on admission, bpm	75.0 ± 14.0	74.0 ± 12.8	74.1 ± 13.8	76.7 ± 15.2	<0.001
GRACE score	129.4 ± 36.7	119.3 ± 34.3	128.2 ± 34.4	140.8 ± 38.1	<0.001
Diagnosis on admission					
STEMI	2062 (34.8)	574 (29.1)	640 (32.4)	848 (43.0)	<0.001
NSTEMI-ACS	3861 (65.2)	1401 (70.9)	1334 (67.6)	1126 (57.0)	
Laboratory results on admission					
D-dimer, ng/mL	125 (73, 230)	57 (39, 73)	125 (104, 148)	328 (230, 518)	<0.001
Fibrinogen, g/L	3.28 ± 0.82	3.13 ± 0.72	3.27 ± 0.76	3.42 ± 0.95	<0.001
Troponin-I, ng/mL	0.41 (0.01, 11.78)	0.9 (0.01, 7.14)	0.21 (0.01, 6.81)	1.43 (0.03, 22.85)	<0.001
Creatinine, umol/l	72 (61, 85)	70 (60, 82)	72 (61, 83)	75 (61, 91)	<0.001
Albumin, g/L	39.5 ± 3.9	40.5 ± 3.6	39.7 ± 3.7	38.3 ± 3.9	<0.001
Hemoglobin, g/L	136.6 ± 16.8	141.1 ± 15.1	136.5 ± 15.9	132.3 ± 18.1	<0.001
Leukocyte count (× 10^9^/L)	8.30 ± 2.99	8.09 ± 2.83	8.17 ± 2.84	8.63 ± 3.25	<0.001
Platelet count (×10^9^/L)	206.6 ± 60.2	206.1 ± 57.1	207.7 ± 59.2	206.0 ± 64.1	0.476
BNP, ng/L	123 (41, 342)	108 (95, 131)	114 (42, 303)	214 (77, 547)	<0.001
Percutaneous coronary intervention details					
Left main disease	527 (8.9)	141 (7.1)	179 (9.1)	207 (10.5)	0.001
Three-vessel disease	1496 (25.3)	413 (20.9)	513 (26.0)	570 (28.9)	<0.001
Use of Intra-aortic Balloon Pump	190 (3.2)	36 (1.8)	57 (2.9)	97 (4.9)	<0.001
TIMI flow grade 0/1 on arrival	4610 (77.8)	1539 (77.9)	1480 (75.0)	1591 (80.6)	<0.001
TIMI flow grade 3 post PCI	5899 (99.6)	1973 (99.9)	1968 (99.7)	1958 (99.2)	0.001
In-hospital Mortality	53 (0.9)	9 (0.5)	6 (0.3)	38 (1.9)	<0.001

*MI* Myocardial infarction, *PCI* Percutaneous coronary intervention, *SBP* Systolic blood pressure, *bpm* Beats per minute, *STEMI* ST-segment elevation myocardial infarction, *NSTEMI-ACS* Non-ST-segment elevation myocardial infarction of acute coronary syndrome, *BNP* Brain natriuretic peptide

### Prognostic performance of D-dimer for the prognosis prediction

When as a continuous variable, D-dimer significantly predicted in-hospital mortality in the univariate Logistic regression analysis (OR: 1.069, 95% CI: 1.046–1.093, *p*<0.001, for per 100 ng/mL increase) (Table 2). After adjusting for covariates, D-dimer was still independently associated with in-hospital mortality: an increased in-hospital mortality risk of 6.0% for per 100 ng/mL increase in D-dimer concentration (OR: 1.060, 95% CI: 1.026–1.094, *p*<0.001) (Table 2).

**Table 2:** Effects of multiple variables on Clinical Outcomes in Univariate and Multivariate Analysis

	Univariate Analysis			Multivariate Analysis		
	OR	95% CI	*p* value	OR	95% CI	*p* value
GRACE score	1.034	1.028–1.041	<0.001			
D-dimer as a continuous variable						
D-dimer per 100 *ng*/mL increase	1.069	1.046–1.093	<0.001	1.060	1.026–1.094	<0.001^a^
D-dimer as a categories variable						
Low D-dimer group	Reference		<0.001	Reference		0.003
Intermediate D-dimer group	0.689	0.245–1.941	0.481	0.668	0.176–2.532	0.553
High D-dimer group	4.362	2.104–9.044	<0.001	3.079	1.079–8.788	0.036^a^

^a^Adjusted for age, History of Hypertension, Dyslipidemia, SBP on admission, Heart rate on admission, Diagnosis on admission, Troponin-I, fibrinogen, creatinine, Albumin, Hemoglobin, Leukocyte count, Platelet count, BNP, Left main disease, Three-vessel disease, Use of Intra-aortic Balloon Pump and TIMI flow grade 3 post PCI;

When categorized into three groups, D-dimer remained significantly predictive of in-hospital mortality (Table 2). In the univariate Logistic regression analysis, High D-dimer group had a substantially higher risk of in-hospital death (OR: 4.362, 95% CI: 2.104–9.044, *p*<0.001), compared with Low and Intermediate D-dimer group (Table 2). In the multivariable Logistic regression analysis, High D-dimer group still had a significantly higher in-hospital mortality (OR: 3.079, 95% CI: 1.079–8.788, *p* = 0.036) (Table 2).

### Prognostic performance of D-dimer, GRACE score and GRACE+ D-dimer for the prognosis prediction

**Fig. 2:**
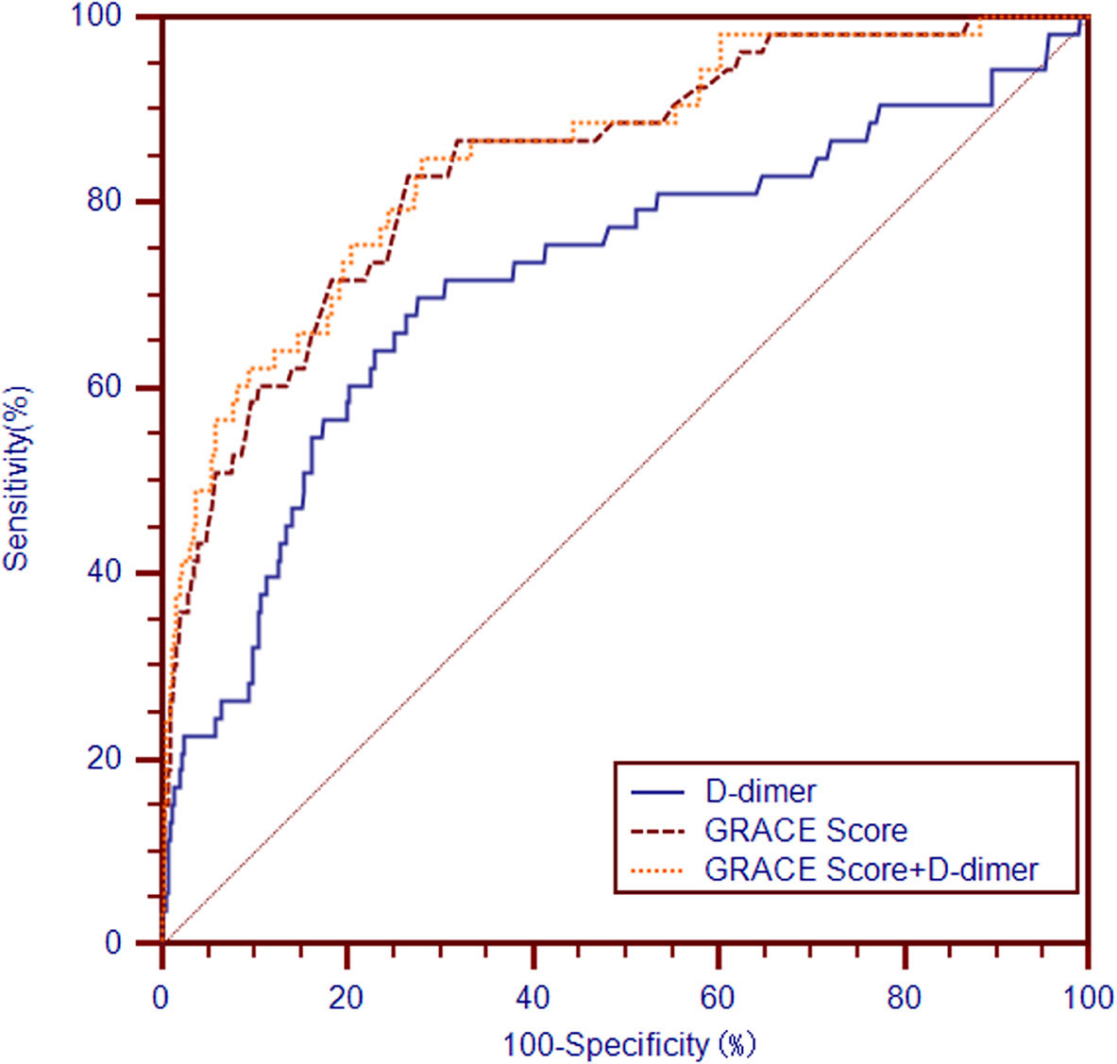
Receiver operating characteristic curves of D-dimer, GRACE and GRACE+D-dimer for in-hospital death prediction

The C-statistic of D-dimer, GRACE score and GRACE score + D-dimer for predicting in-hospital mortality were 0.719 (95% CI 0.708 to 0.731), 0.842 (95% CI 0.833 to 0.851) and 0.851 (95% CI 0.842 to 0.860) (Table 3 and Fig. 2), respectively. The cut-off values for D-dimer was 212 ng/mL with a sensitivity of 0.698 and a specificity of 0.724.

**Table 3:** GRACE, GRACE+ D-dimer and D-dimer performance for the prognosis prediction

	Discrimination				Calibration		Precision
	C-statistic	Standard error	*p* value	95% CI	HL *p*-Value	R^2^	Brier Score
GRACE score	0.842	0.0285	<0.001	0.833–0.851	0.733	0.192	0.0085
D-dimer	0.719	0.0414	<0.001	0.708–0.731	0.002	0.037	0.0089
GRACE+ D-dimer	0.851	0.0285	<0.001	0.842–0.860	0.503	0.208	0.0083

### Improvement of the prognostic performance of GRACE score combining D-dimer

The Hosmer-Lemeshow *p* value of the GRACE score was highest; the Nagelkerke-R^2^ of GRACE +D-dimer was highest; the Brier score of GRACE +D-dimer was lowest (Table 3). The prognostic performance of GRACE +D-dimer was better than GRACE score (C-statistic: *z* = 2.269, *p* = 0.023; IDI: 0.016, *p* = 0.032; NRI: 0.291, *p* = 0.035) and D-dimer (C-statistic: *z* = 3.114, *p* = 0.001; IDI: 0.051, *p*<0.001; NRI: 0.928, *p*<0.001), respectively (Table 4).

**Table 4:** Comparisons of the predictive performance of GRACE, GRACE+ D-dimer and D-dimer for the prognosis prediction

	*z* for C-statistic	*p* for C-statistic	NRI	*p* for NRI	IDI	*p* for IDI
GRACE vs. D-dimer	2.786	0.005	0.941	<0.001	0.045	0.003
GRACE+ D-dimer vs. GRACE	2.269	0.023	0.291	0.035	0.016	0.032
GRACE+ D-dimer vs. D-dimer	3.144	0.001	0.928	<0.001	0.051	<0.001

## Discussion

This study focused on the association between D-dimer and in-hospital mortality in ACS patients undergoing PCI. It found that: (1) D-dimer could independently predict in-hospital mortality; (2) GRACE score + D-dimer got a better prognostic performance than GRACE score, and D-dimer could significantly improve the prognostic performance of GRACE score.

As a kind of soluble degradation product of cross-linked fibrin, D-dimer increased when coagulation was active, thrombin was generated, or fibrin was formed [5]. Pathophysiological factors associated with plasma D-dimer levels in coronary artery disease patients were studied. In stable coronary artery disease subjects, high D-dimer had a significant association with plaque necrosis, lipoprotein (a) and plaque calcium [6]. D-dimer level also independently predicted no-reflow in STEMI patients with primary PCI [7, 8]. A CMR imaging study also found that high D-dimer on admission predicted larger myocardial infarct size, larger area at risk, and smaller myocardial salvage index in STEMI patients undergoing PCI [9]. Moreover, D-dimer might be associated with advanced myocardial injury [9]. Furthermore, the association between higher D-dimer and the clinical long-term adverse outcome other studies was also confirmed. Two studies focused on the role of D-dimer for predicting the prognosis in patients with STEMI receiving PCI [13, 14]. They confirmed that D-dimer could independently predict the long-term mortality in those patients [13, 14]. Other studies also verified and extended this observation. High D-dimer was associated with long-term adverse outcome in stable coronary artery disease [10, 11], and ACS [12]. Our study also found that D-dimer was independently associated with in-hospital mortality in patients with ACS receiving PCI.

Taken together, these findings had clinical value. It may be worth to monitor D-dimer in patients with ACS, which would identify the ACS patients at high risk. Moreover, the LIPID study presented evidence that D-dimer reflected an inflammatory state [11]. The studies also showed that anticoagulant and anti-inflammatory treatments could reduce ischemic events and venous thromboembolism [20–23]. So, high D-dimer patients may benefit from the anticoagulant and anti-inflammatory treatments. Such a strategy was tested in the Attenuation of D-dimer Using Vorapaxar to Target Inflammatory and Coagulation Endpoints (ADVICE) (NCT02394730) in patients with HIV. In the future, adequately powered randomized studies, targeting on attenuation of high D-dimer in ACS patients, should be performed to obtain the conclusions.

The GRACE score is a widely recommended and important prognostic tool in patients with ACS [1–4]. It contains the main traditional risk factors. However, recently, more and more new risk factors, which were not contained by GRACE score, were studied. D-dimer was an important member of them [5]. This study found that GRACE score combining D-dimer showed good discrimination, calibration and precision. The prognostic performance of GRACE score combining D-dimer was also better than GRACE score. The prognostic performance of the GRACE score could be significantly improved by D-dimer. With the help of the new model (GRACE score combining D-dimer), more accurate assessment of the in-hospital mortality risk and better clinical decisions in patients with ACS will be made.

There were some limitations in this study. Firstly, it was a single-center, prospective and observational study. It was hard to completely adjust potential confounders and selection bias. Secondly, high-sensitivity C-reactive protein, other proinflammatory cytokines, or markers of oxidative stress were not included in this study. However, the LIPID study confirmed that, even after adjustment for high-sensitivity C-reactive protein, D-dimer was still a significant predictor [11]. Thirdly, the use of unfractionated heparin or low molecular weight heparin might affect D-dimer levels. Fourthly, some high-risk patients might be excluded because of the use of unfractionated heparin or LMWH, which might cause the potential selection bias. At last, data about the history of medication treatment that influenced coagulation and inflammatory, such as statin, was not complete. Patients with the high coagulation and inflammatory status could benefit from the statin use [24].

## Conclusion

D-dimer was an independent predictor of in-hospital mortality in patients with ACS undergoing PCI. The prognostic performance of GRACE score combining D-dimer was better than the GRACE score. D-dimer could significantly improve the prognostic performance of the GRACE score.

## Supplementary information

**Additional file 1: Table S1.** Effects of multiple variables on in-hospital mortality in Univariate Analysis.

## Data Availability

The datasets used and/or analyzed during the current study are available from the corresponding author on reasonable request.

## Abbreviations

ACS: Acute coronary syndrome
BNP: Brain natriuretic peptide
bpm: beats per minute
CIs: Confidence intervals
GRACE score: The Global Registry of Acute Coronary Events score
HL: The Hosmer-Lemeshow test
IDI: Integrated discrimination improvement
LMWH: Low molecular weight heparin
MI: Myocardial infarction
NRI: Net reclassification improvement
NSTEMI-ACS: Non-ST-segment elevation myocardial infarction of acute coronary syndrome
PCI: Percutaneous coronary intervention
PCI: Percutaneous coronary intervention;
SBP: Systolic blood pressure
STEMI: ST-segment elevation myocardial infarction
