# Supplementary material for: Hospital mortality in acute coronary syndrome: adjustment of GRACE score by D-dimer enables a more accurate prediction in a prospective cohort study
Source: BMC Cardiovasc Disord. 2019 Nov 10;19:252. doi: 10.1186/s12872-019-1239-4 (PMC6842504; doi:10.1186/s12872-019-1239-4)
Supplement: Supplementary file 1 — Additional file 1: Table S1. Effects of multiple variables on in-hospital mortality in Univariate Analysis. [file 12872_2019_1239_MOESM1_ESM.doc]

**Table S1 Effects of multiple variables on in-hospital mortality in Univariate Analysis.**

|  | **OR** | **95% CI** | **P** |
| --- | --- | --- | --- |
| **Age, yrs** | **1.077** | **1.048-1.106** | **＜0.001** |
| **Gender** | **1.077** | **0.603-1.923** | **0.801** |
| **History of Diabetes Mellitus** | **1.111** | **0.628-1.967** | **0.717** |
| **History of Hypertension** | **0.608** | **0.354-1.045** | **0.072** |
| **History of MI** | **1.218** | **0.518-2.861** | **0.651** |
| **Dyslipidemia** | **0.581** | **0.338-1.001** | **0.051** |
| **Prior PCI** | **0.504** | **0.157-1.622** | **0.251** |
| **SBP on admission, mm Hg** | **0.967** | **0.954-0.981** | **＜0.001** |
| **Heart rate on admission, bpm** | **1.033** | **1.018-1.048** | **＜0.001** |
| **Diagnosis on admission** | **3.251** | **2.078-5.086** | **＜0.001** |
| **Fibrinogen, g/L** | **1.789** | **1.397-2.291** | **＜0.001** |
| **Troponin-I, ng/mL** | **1.017** | **1.011-1.024** | **＜0.001** |
| **Creatinine, umol/l** | **1.023** | **1.016-1.029** | **＜0.001** |
| **Albumin, g/L** | **0.877** | **0.821-0.935** | **＜0.001** |
| **Hemoglobin, g/L** | **0.974** | **0.959-0.989** | **0.001** |
| **Leukocyte count (×10^9^/L)** | **1.245** | **1.173-1.321** | **＜0.001** |
| **Platelet count (×10^9^/L)** | **0.995** | **0.990-1.000** | **0.059** |
| **BNP, ng/L** | **1.001** | **1.001-1.001** | **＜0.001** |
| **Left main disease** | **3.387** | **1.800-6.373** | **＜0.001** |
| **Three-vessel disease** | **2.117** | **1.222-3.667** | **0.007** |
| **Use of Intra-aortic Balloon Pump** | **24.087** | **13.656-42.486** | **＜0.001** |
| **TIMI flow grade 0/1 on arrival** | **0.716** | **0.349-1.471** | **0.363** |
| **TIMI flow grade 3 post PCI** | **0.060** | **0.017-0.207** | **＜0.001** |

**MI, myocardial infarction; PCI, percutaneous coronary intervention; SBP, systolic blood pressure; bpm, beats per minute; BNP, brain natriuretic peptide**
